# Supplementary material for: Resolving the full spectrum of human genome variation using Linked-Reads
Source: Genome Res. 2019 Apr;29(4):635–45. doi: 10.1101/gr.234443.118 (PMC6442396; doi:10.1101/gr.234443.118)
Supplement: Supplemental Material [file supp_gr.234443.118_Supplemental_longranger-2.2.zip › longranger-2.2.tar.bz2!/lr2.2-public/lib/stan/cmdstan/stan/doc/api/html/agrad_2rev_2functions_2step_8hpp_source.html]

Stan: src/stan/agrad/rev/functions/step.hpp Source File


|  |  |
| --- | --- |
|  | Stan  2.5.0  probability, sampling & optimization |


- Main Page
- Related Pages
- Namespaces
- Classes
- Files

- File List
- File Members

All Classes Namespaces Files Functions Variables Typedefs Enumerations Enumerator Friends Macros Pages

- src
- stan
- agrad
- rev
- functions

step.hpp

Go to the documentation of this file.

1 #ifndef STAN\_\_AGRAD\_\_REV\_\_FUNCTIONS\_\_STEP\_HPP

2 #define STAN\_\_AGRAD\_\_REV\_\_FUNCTIONS\_\_STEP\_HPP

3

4 #include <stan/agrad/rev/var.hpp>

5

6 namespace stan {

7  namespace agrad {

8

25  inline var step(const stan::agrad::var& a) {

26  return var(new vari(a.vi\_->val\_ < 0.0 ? 0.0 : 1.0));

27  }

28

29  }

30 }

31 #endif

stan::agrad::vari::val\_

const double val\_

The value of this variable.

**Definition:** vari.hpp:37

stan::agrad::var::vi\_

vari \* vi\_

Pointer to the implementation of this variable.

**Definition:** var.hpp:40

stan::agrad::vari

The variable implementation base class.

**Definition:** vari.hpp:28

var.hpp

stan::agrad::var

Independent (input) and dependent (output) variables for gradients.

**Definition:** var.hpp:27

stan::agrad::step

var step(const stan::agrad::var &a)

Return the step, or heaviside, function applied to the specified variable (stan). ...

**Definition:** step.hpp:25


---

[ Stan Home Page ]

*© 2011–2014,
Stan Development Team.*
